# Long Non-Coding RNAs: Discoveries, Mechanisms, and Research Strategies in Seeds

**DOI:** 10.3390/genes14122214

**Published:** 2023-12-14

**Authors:** Xiumei Li, Qinjian Liu, Jun Liu

**Affiliations:** Guangdong Provincial Key Laboratory for Crop Germplasm Resources Preservation and Utilization, Agro-Biological Gene Research Center, Guangdong Academy of Agricultural Sciences, Guangzhou 510640, China; lixiumei@agrogene.ac.cn (X.L.); aeo22@126.com (Q.L.)

**Keywords:** seed development, seed germination, lncRNA, characterization, interaction

## Abstract

Seeds provide nutrients for the embryo and allow for dormancy in stressed environments to better adapt the plant to its environment. In addition, seeds are an essential source of food for human survival and are the basis for the formation of food production and quality. Therefore, the research on the genetic mechanism of seed development and germination will provide a theoretical basis and technical support for the improvement of crop yield and quality. Recent studies have shown that long non-coding RNAs (lncRNAs) occupy a pivotal position in seed development and germination. In this review, we describe the key processes in seed biology and examine discoveries and insights made in seed lncRNA, with emphasis on lncRNAs that regulate seed biology through multiple mechanisms. Given that thousands of lncRNAs are present in the seed transcriptome, characterization has lagged far behind identification. We provide an overview of research strategies and approaches including some exciting new techniques that may uncover the function of lncRNAs in seed. Finally, we discuss the challenges facing the field and the opening questions. All in all, we hope to provide a clear perspective on discoveries of seed lncRNA by linking discoveries, mechanisms, and technologies.

## 1. Introduction

Seed, as a carrier of genetic diversity, is not only the basis of agricultural production but also an essential material for the long-term preservation of germplasm resources and species diversity. In an agronomic sense [1], the term “seed” is the unit of reproduction that is capable of developing into a single plant, including any type of planting material that can be used in producing a crop, i.e., sexual and asexual formation of seeds (apomictic seed), as well as vegetative organs (tissues of the dominant sporophytic generation) propagating offspring and artificial seeds cultivated by tissue culture techniques. Seeds are either developed from sexual reproduction or apomixis. Authentic seed refers to specific propagules of gymnosperms and angiosperms, which are formed from an ovule by pollination and fertilization, whereas apomixis refers to a set of reproductive mechanisms that avoid meiosis and egg cell fertilization to generate clonal seeds [2]. Nevertheless, given that most studies on seeds focus on sexual reproduction and agamospermy, vegetative organs and artificial seeds are not within the scope of our consideration.

Seeds consist of three main structures: embryo, endosperm, and seed coat, which are developed from a zygote (fertilized egg) or somatic cells, primary endosperm nucleus (fertilized polar nucleus), and integument. The process of seed formation and germination is accompanied by the temporal and spatial expression of specific genes, which is regulated by multiple physiological signals in vivo and environmental signals, such as light, temperature, and hormones. Seed development and germination are key physiological phases that contribute to seed generation, survival, and dispersal, as well as agricultural production [3]. However, the regulatory network and mechanism in seed development and germination are vague. In-depth research on the molecular mechanisms regulating seed development and germination can be applied to genetic modification, which will help to improve seed yield, quality, and vitality.

In the last two decades, advances in high-throughput nucleic acid sequencing have revealed that most eukaryotic genomes are transcribed, and the vast majority do not encode proteins. Since realizing the regulatory roles of the “junk DNA”, efforts to identify protein non-coding RNA (ncRNA) molecules have greatly accelerated, thanks to comprehensive technology development that has enabled their identification and characterization. These ncRNAs are initially recognized as transcriptional noise because of poor sequence conservation and low abundance of transcripts, including long non-coding RNAs (lncRNAs), microRNAs (miRNAs), small interfering RNAs (siRNAs), small nuclear RNAs (snRNAs), ribosomal RNAs (rRNAs), transfer RNAs (tRNAs), and circular RNAs (circRNAs) [4,5]. A recent study demonstrated that lncRNAs are associated with domestication-related seed traits [6] and seed germination [7]. Many seed traits, like color, size, shape, vigor, nutritional content, and dormancy formed in seed development, determine the new cycle of plant growth from seed germination. However, in many cases, the seed traits exhibit negative correlations due to the trade-offs involved. Encouragingly, the contradiction between “high yielding” and “early maturing” was overcome in rice through an *Ef-cd* (early flowering-completely dominant) lncRNA. *Ef-cd* may recruit an undefined complex, which causes an increase in the H3K36me3 level in the *OsSOC1* locus and promotes the *OsSOC1* transcript [8]. This gives us a clue that lncRNA could be a vital contributor to the fine regulation of paradox traits, and the lncRNA locus may be a valuable genetic resource for crop breeding. Here, we review the current studies of lncRNAs that participated in the regulation of seed development and seed germination, as well as the application of approaches and strategies for characterizing lncRNA.

## 2. The Mechanisms of Action of lncRNA

LncRNAs are usually RNA molecules with a transcript length of more than 200 nucleotides and have little or no coding ability. Most lncRNAs, similar to mRNAs, can be transcribed by RNA polymerase II, capped at the 5′ end, polyadenylated at the 3′ end, and splicing [9]. In plants, some lncRNAs are transcribed by plant-specific RNA polymerases IV and V [10]. LncRNAs are mainly distributed in the nucleus and cytoplasm, while the 5′ cap structure and 3′ polyadenylation are mainly completed in the nucleus. LncRNAs are abundant in organisms, e.g., 173,112 lncRNA transcripts in the human body tissues (http://www.noncode.org/analysis.php (accessed on 13 December 2023)), compared with 13,599 in *Arabidopsis thaliana* lncRNA prediction transcript (https://www.tobaccodb.org/plncdb/ (accessed on 13 December 2023)). There are many types of lncRNAs. With respect to the position of lncRNAs in the genome with protein-coding genes, they can be classified into intronic lncRNAs, intergenic lncRNAs, promoter lncRNAs, enhancer lncRNAs, natural antisense lncRNAs, sense lncRNAs, bidirectional lncRNAs, non-poly(A) lncRNAs, and small nucleolar RNA-ended lncRNAs (sno-lncRNAs) [11,12]. Protein-coding genes are usually located on one of the strands of DNA, and lncRNAs transcribed from the complementary strand will have some complementary base pairing regions with the protein-coding genes, so lncRNAs are involved in regulating the expression of protein-coding genes as a silencer or enhancer [13]. Approximately 50% of mRNA transcripts are distributed over 1000 bp in length, whereas lncRNAs have a shorter exon length and lower exon number, and about 70–80% of lncRNA transcripts range in length from 200–1000 bp, and 20–30% of lncRNA transcripts are longer than 1000 bp [14]. In addition, most lncRNAs have high tissue specificity, less sequence conservation, and low expression.

As a growing number of lncRNAs have been revealed to participate in development and stress resistance, they have diverse biochemical mechanisms that regulate gene expression by affecting transcription, post-transcriptional mRNA stability, translation, and epigenetics. They may activate or inhibit gene transcription either locally or remotely. The effect of lncRNA on mRNA in the vicinity of the same chromosome is called cis-regulation, while the effect on long-distance mRNA is called trans-regulation. LncRNAs also act as bait, scaffold, guide, endogenous target mimics, and enhancers, and it has been found that there are commonalities in the mechanisms of action in plants and animals, which can be summarized into eight categories. When lncRNA is located in the upstream promoter region of the coding gene, it interferes with the expression of downstream mRNA by initiating transcription (1) or modifying chromatin (2). When lncRNA and mRNA are reverse complementary, mRNA degradation can be caused by interfering with the splicing form of mRNA (3) or generating miRNA, PIWI-interacting (piRNA), and other small molecular RNA (4). When lncRNA binds to specific proteins, it can regulate the activity of corresponding proteins (5) and cell localization (6) or form complexes with proteins as structural components (7), thereby affecting mRNA transcription or translation. Some abundant lncRNAs affect gene expression by functioning as competitive endogenous RNA (target-mimic for miRNA and avoids cleavage of its target mRNA) (8), thus affecting the stability of mRNA. LncRNAs were previously defined as having little or no protein-coding potential. However, as research has progressed, a handful of lncRNAs have been found to translate micropeptides and perform biological functions using these encoded micropeptides.

## 3. The Biological Roles of lncRNAs in Seed Biology

During sexual reproduction in angiosperms, two sperm from pollen fuse with egg cells and two polar nuclei in the embryo sac to form a diploid zygote and a triploid nucleus. This double fertilization involving two male nuclei can also occur in gymnosperms, but fusion between haploid nuclei does not result in a triploid endosperm. Most prominently in the gymnosperms, only one pollen sperm cell is released from the microspore, but several egg cells are present in the female gametophyte, each of which may be fertilized. The cooperative regulation of maternal and zygotic tissue affects seed growth and final size. After the seed matures, it enters a dormant state, and germinates until the external environment becomes suitable for plant growth. Therefore, the three basic seed structures, embryo, endosperm, and seed coat, interact closely in seed development and disintegrate gradually in seed germination, along with the regulation of gene expression, cell division, and nutrient supply.

### 3.1. Seed Development

#### 3.1.1. Embryogenesis and Endosperm Development

Embryogenesis includes cell division, differentiation, organogenesis, and dormancy establishment, accompanied by the production of the endosperm and the seed coat. Embryo formation can be initiated from either a zygote following gametic fusion (zygotic embryogenesis, ZE) or an asexual embryo that originated from somatic cells (somatic embryogenesis, SE), which is usually induced in vitro [15,16]. The fundamental biological processes involved in the regulation of early embryonic development include zygote activation, embryo polarity establishment, embryo pattern formation, and cotyledon formation, producing simple versions of miniature plants consisting of only the most basic features such as the precursors for all the primary tissues and organs [16]. By comparing the lncRNA profiles of the gametes with that of their offspring zygotes, it is possible to identify newly generated lncRNAs immediately after zygote activation. As early as more than a decade ago, studies have revealed that lncRNAs are essential for controlling cell differentiation and maintaining pluripotency in animal stem cells [17]. However, similar evidence has yet to be described in plant cells, and no lncRNA-related research has been reported in ZE. Several reports described that lncRNAs participate in SE and may form a regulatory network with mRNAs and miRNAs [18]. For instance, differentially expressed longan lncRNAs were involved in expression regulation at each SE stage [19]. In white spruce, stress-induced SE positively regulates the stress response, auxin signal transduction, and target genes related to early SE development. These lncRNAs might have a protective or spongy effect on mRNA targeted by miRNAs and thus participate in SE and ultimately promote embryogenesis in mature somatic embryos [20]. Notably, research on embryo-free seeds of Arabidopsis demonstrated that endosperm development from initiation to degeneration is an autonomous programmed process independent of embryogenesis and is critical for embryo and seed coat development [21]. Then, endosperm development occupies a dominant position in seed development, breaking the misconception of the central role of embryo.

Endosperm development generally goes through an early period of nuclear division and syncytium formation, followed by a process of cellularization. The triploid endosperm, which contains one copy of the paternal genome and two copies of the maternal genome, is found only in the angiosperms, making it an excellent system for discovering new features of lncRNAs that are difficult to find in other tissues [22]. For example, imprinting, an epigenetic phenomenon that renders alleles differentially expressed depending on their parental origin, primarily occurs in the endosperm [23,24,25,26,27], and a few genes are found in the embryo [23,28]. Imprinted lncRNA loci in the intergenic regions were associated with maternally expressed genes and paternally expressed genes that regulate seed traits. Functional analysis indicates that mutations of maternally expressed genes lead to smaller seeds, whereas mutations of paternally expressed genes result in seed sterility [29]. By detecting SNPS between parents and hybrid endosperm tissues, there were some biases in the expression of allelic lncRNAs that were partially dependent on the parental effect, and the expression of most lncRNAs was not dosage-sensitive [22]. A few years later, MISSEN, the first lncRNA identified as a regulator of endosperm development in rice, is parent-of-origin and expressed in endosperm. Overexpression plant lines exhibited shriveled seeds with abnormal cytoskeleton polymerization because *MISSEN* regulates tubulin function by hijacking a helicase family protein (HeFP) during nucleus division and endosperm cellularization. In wild-type seeds, its expression was inhibited precisely by histone H3 lysine 27 trimethylation (H3K27me3) modification after pollination [30]. 

**Figure 1:**
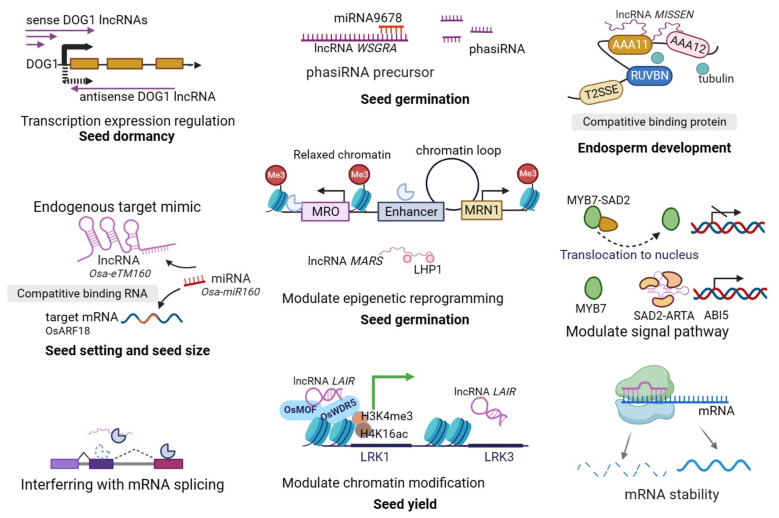
Mechanisms of action of lncRNA-mediated seed biology. In “Seed dormancy”, purple arrows represent the transcriptional direction of lncRNA, the black arrow is the *DOG1* transcript direction, and the black thick solid arrow indicates that the sense lncRNA enhance the expression of *DOG1* while the black thick dotted arrow indicates that the antisense lncRNA reduce the expression of *DOG1*. In “Seed setting and seed size”, the arrows indicate that both Osa-eTM160 and OsARF18 have complementary sites with Osa-miR160. In “mRNA stability”, the arrows indicate that lncRNA binds to the mRNA to increase or reduce the stability of mRNA. In “Modulate signal pathway”, the dotted arrow indicates that ARTA impairs SAD2-mediated nuclear trafficking of MYB7 in plant responses to ABA, SAD2 interacts with ARTA through its lncRNA binding region. Otherwise, arrow with slashes means inhibiting expression and arrow without slashes means prompting expression.

The stored reserves in seeds are present in both embryonic and extraembryonic tissues in different proportions. For instance, in most cereals, the major starch and protein occur in the endosperm, and the oil is present in the embryo (scutellum). In rare cases, the nucellus is retained to become the primary storage tissue (e.g., perisperm), providing a nutrient source for the growing seedling in angiosperms. The persistent megagametophyte takes on this responsibility in the gymnosperms [31]. The endosperm may be surrounded by an aleurone layer and accumulates nutrients as a permanent storage tissue in endospermic seeds. In contrast, cotyledons become the major storage tissue in non-endospermic seeds since the developing embryo occludes endosperm, and then stored reserves are reorganized in the cotyledon. These non-endospermic embryos must contain oils, carbohydrates, and proteins necessary for seed germination and seedling development. The quality and yield of the storage reserves are regulated by the biosynthetic pathway and environmental conditions during seed development. However, the studies related to environmental factors such as abiotic stress in seed development focus on protein-coding genes. Even if there are studies on lncRNA, they are mainly in flower or vegetable organs. LncRNAs identified from barley, wheat, soybean, sunflower, rapeseed, and other plants seem to be closely related to the biosynthesis, transport, and metabolism of stored reserves in seeds (review in [32]). For instance, lncRNAs identified in maize exhibit spatio-temporal specificities from the embryo and three cell types of endosperms [33,34]. In addition, a few studies have found that lncRNAs are involved in seed size and weight, influencing seed filling and yield [6,35]. Although the phenotype and expression of genes controlling grain size and weight are altered in RNA interference plants, the accurate regulation mechanisms remain to be further studied. One reasonable speculation derived from studies of *Setaria italica* and chickpea is that lncRNAs related to grain yield act as miRNA target mimics and regulate expression of targets by competing for the interaction between miRNAs and their target mRNAs [36,37] (Figure 1). In fact, a few years ago, researchers demonstrated that the role of a rice lncRNA acts as an endogenous miRNA target mimic (eTM), in which *osa-eTM160* attenuated the repression of *osa-miR160* on *osa-ARF18* mRNAs during early anther developmental stages, thereby affecting rice seed set and seed size [38]. Therefore, lncRNA is associated with the auxin signal during seed development, which controls the fates of embryonic cells and endospermic cells in the embryogenesis and maturation stages. Another mechanism by which the lncRNA plays an active role in seed development is the activation of genes by forming complexes with epigenetic modification proteins. *LAIR*, a lncRNA transcript in the antisense strand of the neighboring gene *LRK* (leucine-rich repeat receptor kinase) cluster, interacted with histone modification proteins OsMOF and OsWDR5, leading to enrichment of histone marks (H3K4me3 and H4K16ac) and activation of *LRK1* and UTRs of *LRK1* [39]. Some lncRNAs may be cleaved by a miRNA to produce non-coding phased small interfering RNA (phasiRNA), and loci that generate phasiRNA are known as PHAS loci in either protein-coding or non-coding genome regions. It has been reported that a long hairpin structure RNA (LHR) is a phasiRNA precursor. A T-DNA insertion *lhr* mutant with destruction of the hairpin structure completely abolished the production of the phasiRNA and reduced grain size and weight [40].

#### 3.1.2. Seed Dormancy and Seed Longevity

Longevity and dormancy depth are determined in the maternal plants during seed development [41]. During maturation, seed undergoes a series of changes that terminate development, the accumulation of stored reserves gradually stops, the water content declines, the protoplasm changes from the sol state to gel state, the respiration rate decreases to the lowest level, and the embryo enters a metabolically quiescent state. Dormancy, which many species have acquired, is also established in this stage. The absence of dormancy in cereal species could result in precocious germination on the maternal plants, such as pre-harvest sprouting. The well-known gene Delay of Germination 1 (DOG1) is a main QTL controlling seed dormancy in Arabidopsis, showing reduced seed dormancy in T-DNA insertional mutants [42]. So far, it has reported that DOG1 transcription is regulated by multiple mechanisms, including repression by the antisense lncRNA (asDOG1) *in cis* [43,44] and enhancement by a variety of sense lncRNAs (PUPPIES) on the promoter of *DOG1* [45], as well as alternative polyadenylation (APA) generating short DOG1 (shDOG1) and long DOG1 (lgDOG1) isoforms [46]. In addition, DOG1 is an exosome-sensitive PROMPT, in which RNA polymerase II initiates transcription bidirectionally from gene promoters, producing sense pre-mRNAs on the forward strand and promoter upstream unstable RNAs on the reverse strand [47]. Seed dormancy significantly decreases under a high-temperature environment during seed maturation. The expression patterns of five wheat lncRNAs exhibited a high fold change after high-temperature treatment, implying their critical roles in high temperature-mediated dormancy [48], while DOG1 is a low temperature-regulated gene. 

Dormancy and longevity are critical adaptive traits that contribute to seed lifespan, but the discoveries on their relationship are conflicting [49]. On the one hand, the loss-of-function mutant in the DOG1 gene exemplified the hypothesis that they are positively correlated. On the other hand, Germination Ability After Storage (GAAS) loci co-located with DOG genes, and dormancy and longevity are negatively correlated for the QTLs [50]. Of the many factors influencing seed longevity during drying and subsequent handling, the top two factors are seed moisture content (or equilibrium RH) and environmental temperature. The combination of high humidity and high temperature has been used in “controlled deterioration” tests (CDT) or “accelerated aging” to reduce seed longevity significantly, even though CDT does not entirely mimic natural aging [50]. Many of the genes that have been identified as being potentially relevant for seed longevity are related to DNA repair mechanisms [51,52,53], ABSCISIC ACID-INSENSITIVE3 (ABI3) [54,55], and the reactive oxygen species metabolic process [56]. The research about the seed longevity of lncRNAs is still in its infancy, mainly in the extraction, prediction, and identification, but not in the functional verification and mechanism. Although seed aging is a common physiological and irreversible phenomenon in the storage process, which begins to occur immediately after the seed reaches its vitality peak, the effect of aging stress on the seed can be observed and determined after long-term storage by a germination assay. Different species of seeds have various store reserves in different proportions, as discussed above, increasing the difficulty and complexity of research on seed longevity. Therefore, the aging rate results from the joint action of genetic factors and the natural environment. In addition, artificial aging is likely to mask some of the molecular mechanisms underlying natural aging. With the continuous research of protein-coding genes on seed longevity in more detail, the investigation of lncRNA will be further advanced. A genomic view of lncRNAs in rice seed revealed that lncRNAs undergo extensive alternative splicing during the transition from milk seed to mature embryo and endosperm, and lncRNAs could maintain more exons in embryos [57]. Alternative splicing forms of a rice lncRNA LNC_037529 were also identified in artificial aging seed [58]. These results suggest that the alternative splicing of lncRNA is widely present in metabolic processes. 

### 3.2. Seed Germination

The likely effects of seed quality traits on seed vigor are determined during seed ripening and assure seed longevity and germination in different environmental conditions. It is well known that the plant hormones abscisic acid (ABA) and gibberellin (GA) are the primary hormones that antagonistically regulate seed dormancy and germination [59,60]. Several studies conducted in the last decade have found that lncRNAs modulate the GA/ABA signaling and seed germination. A lncRNA BoNR8 processed by RNA polymerase III in cabbage possibly relates to ABA-responsive genes. Overexpression of BoNR8 in Arabidopsis plants leads to decreased germination rates, less primary root elongation, and incomplete silique development. Accordingly, the expression of ABA-related genes was changed in the overexpression lines [61]. However, whether BoNR8 competes with its Arabidopsis homologous AtNR8 is still unknown, nor is the mechanism of how *BoNR8* interacts with ABA-responsive genes. Guo and colleagues provided evidence that lncRNA WSGAR is targeted by wheat-specific miR9678 to trigger phasiRNA production. This miR9678, expressed specifically in the scutellum during seed development and germination, is negatively associated with seed germination. ABA signaling proteins bind the promoter of miR9678 precursor to activate its expression, and overexpression of miR9678 reduces the bioactive GA level. Nevertheless, direct RNA cleavage mediated by WSGAR-derived phasiRNA is not the reason for the transcriptome changes in miR9678 overexpression lines [62], leaving unknown phasiRNAs and their related function. It has been proposed that lncRNAs can modulate the transcriptional activity of adjacent genes by shaping local three-dimensional (3D) chromatin conformation [63,64]. Functionally related genes are either scattered or clustered in the genome, and clustered features allow the transcription to be controlled coordinately. For example, marneral, one of four triterpene scaffolds, is governed by enzymes encoded by genes organized in clusters. LncRNA MARS localizes inside the Arabidopsis marneral cluster, which decoys LIKE HETEROCHROMATIN PROTEIN 1 (LHP1) away from the marneral and promotes the formation of a chromatin loop. The MARS-mediated chromatin loop brings the MARNERAL SYNTHASE 1 (MRN1) promoter and a distal ABA-responsive enhancer together to dynamically regulate MRN1 transcriptional activation, thus affecting seed germination in response to ABA [65]. 

Recently, the roles of lncRNAs in seed germination via crosstalk with hormones and environment have also been elucidated in Arabidopsis. *HIDDEN TREASURE 1* (*HID 1*) was identified as a repressor of ABA biosynthesis and a positive regulator of phytochrome B (phyB) dependent seed germination within 48 h of imbibition. It directly inhibits 9-CIS-EPOXYCAROTENOID DIOXYGENASE (NCED9) at the transcriptional level by interacting with ARABIDOPSIS TRITHORAX-RELATED7 (ATXR7), an H3K4me3 methyltransferase, resulting in decreasing occupancy of ATXR7 and H3K4me3 modification at the NCED9 locus [7]. Additionally, a lncRNA from *MtCIR1 Medicago truncatula* renders seed germination more sensitive to salt stress by suppressing the expression of the ABA catabolic enzyme CYP707A2 and ABA signaling during seed germination [66].

## 4. Available Plant lncRNA-Dedicated Research Tools

### 4.1. Sampling of Specific Cell Types in Seed

To understand seed development and germination processes from a global perspective, researchers need to isolate and collect sufficient specific cell types and tissues, but this is a challenge and inaccessible due to their small size and embedding in maternal fruit and seed tissues [67]. Many efforts have been made to develop different approaches, such as manual dissection, laser capture microdissection (LCM), fluorescent-activated nuclei sorting (FANS), and isolation of nuclei tagged in specific cell types (INTACT) to obtain transcriptome data in embryo and endosperm from Arabidopsis to crop plants. In maize, endosperm comprises three distinct types of tissues, including the starchy endosperm, the basal endosperm transfer cell layer, and the aleurone cell layer. LCM and its optimal protocol for maize kernels have been developed specifically to access transcript profiling of the early stages of endosperm development [68,69]. The cryo-dissection method was used to isolate tissues and identify lncRNAs from developing endosperm, although it is rather labor-intensive [70]. The advantage of LCM and cryo-dissection is that producing transgenic plants is dispensable, as the targeted tissues and cells can be isolated from the heterogeneous tissue under direct microscopic visualization without transgenic labeling. Unfortunately, only parts of the cell can be effectively collected from tissue sections, with possible tissue contamination and low RNA quality. The strategy of FANS is to label nuclei with GFP driven by cell-type specific promoters which are only active either in the cells or the tissue, and GFP-positive nuclei are sorted by flow cytometry. This approach avoids generating protoplasts in large amounts, applying to Arabidopsis embryos [71] and endosperm [72]. Unlike FANS, INTACT affinity labels the nuclei by genetically expressing biotinylated nuclear envelope proteins in the target cell type, and then tagged nuclei at high purity can be isolated in large quantities using streptavidin-coated magnetic beads. Purified nuclei tagged in specific cell types have been used for RNA-seq [73,74], ChIP-seq, bisulfite-seq [75], and high-throughput chromatin conformation capture (Hi-C) [76] in embryo and endosperm. However, the specificity of the nuclei purified in FANS and INTACT is limited by the expression pattern of the promoter [77].

Additionally, the requirement for transgenic manipulation is unfriendly to plants without transgenic systems. As a result, these two techniques have not been widely applied to other plant species by isolating protoplasts and nuclei. Researchers have employed FANS to construct a gene expression atlas of Arabidopsis early embryos and endosperm at single-cell resolution [78,79,80]. Single-cell RNA (scRNA) sequencing is being developed and applied to non-model species. It is worth noting that known marker genes for most plant tissue types and plant species are relatively limited compared with those for Arabidopsis root [81]. Moreover, current scRNA sequencing is mainly 3′ amplification, which is unable to analyze the alternate splicing and non-polyadenylated RNA. The scRNA sequencing with full-length amplification under development is expected to solve these problems, and applying scRNA sequencing to seed lncRNA makes it possible to identify putative transcription factors orchestrating the cell type-specific lncRNA expression in embryo and endosperm.

### 4.2. Identification, Isolation, and Quantification of lncRNAs

Large-scale identification of lncRNAs in genomes is the most effective way to find new lncRNAs. Common methods for lncRNA identification include RNAseq, microarray, cDNA EST, etc. RNA-seq data sets have shown that a substantial amount of transcriptome within the plant genome benefit from high-throughput sequencing technologies. For example, lncRNAs in the antisense of the protein-coding loci are revealed by analysis of strand-specific direct RNA sequencing-based mapping of polyadenylation sites in the genome [44]. The lncRNAs can be identified when researchers search for the target of miRNA by interrogating the sequencing database, such as the wheat seed germination-associated RNA [62]. Many public ncRNA databases are divergent according to information source, species, type of structure, and the mechanisms for information retrieval. 

There is an urgent demand to develop in silico approaches to recognize the lncRNA from the flood of transcriptome data. However, the computational identification of lncRNAs from massive data is a challenging task due to the series of filtering steps involved. The key signatures of these computational methods are algorithm models and selected features of the transcripts. Reliable algorithm models are required to support machine learning and thus explore the intrinsic characteristics of lncRNA for classification. In turn, selected features impact the model performance in terms of the accuracy and specificity of prediction output. The selected features accumulated gradually, from the initial ORF length and coverage to conservative ratings such as substitution rates and phylogenic scores, to nucleotide composition such as GC content and k-mer, to structural features and epigenetic information. Because the nature of features may give different weights to the lncRNA identification, most developed identification methods adopt multiple features to optimize the accuracy and specificity of prediction results. Nevertheless, a moderate selection of features needs to be considered, as “over-features” would cause the model to overestimate the impact of certain aspects of the feature and significantly reduce the model recapitulation and prediction performance [82]. Additionally, some of the identification tools are alignment-based, which is a way to compare unidentified sequences with protein-coding data to characterize the coding potential of transcript regions. It is not friendly for non-model organisms without well-established genome information and high-quality protein databases. A supervised ensemble machine learning classifier has been used to predict and rank candidate lncRNA based on a training data set of empirically validated lncRNA for future functional validation, in contrast to training data sets of primarily non-validated, non-coding transcripts on animal systems previously [83]. 

Identification of RNA based on in silico analysis should be validated by experiment, which is probably one of the major challenges of RNA research for the next 20 years [84]. For lncRNAs, the first one discovered in Medicago plants, differentially expressed in spontaneous nodules and root, was screened out from 200,000 phages of a cDNA library [85]. ENOD40 was detected using a Northern blot, the oldest and golden standard method for validating and quantifying lncRNA. Moreover, 5′-RACE (rapid amplification of cDNA ends) and 3′-RACE assays are the canonical approaches for isolating lncRNAs containing the 5′ cap and polyadenylated 3′ end. Real-time quantitative PCR, in situ hybridization, and lncRNA promoter-driving GUS reporter lines are broadly used to validate the expression pattern of lncRNA as a control for the high-throughput results. Generally, in situ hybridization and reporter lines are required to annotate the top high-variance genes in cell clusters reliably derived from single-cell transcriptomes, especially for tissues and organisms lacking established marker genes [81]. 

### 4.3. Subcellular Localization of lncRNAs and Interaction with Other Molecules

**Figure 2:**
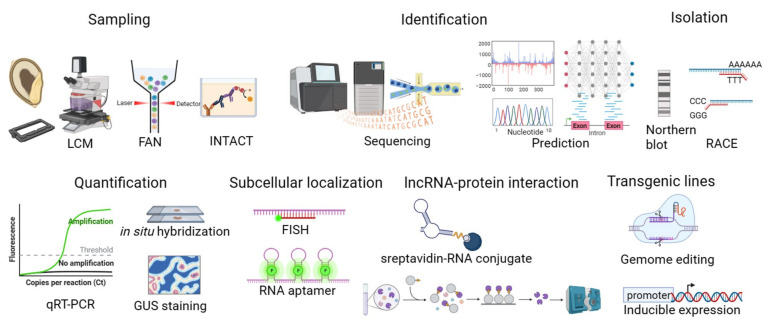
Research strategies of lncRNAs.

RNA subcellular localization is bound up with its biogenesis, processing, and function and also determines the fate and polarity of cells [86]. Many well-studied lncRNAs are prone to reside in the nucleus and regulate gene expression by associating with chromatin [87]. However, some lncRNAs from humans and plants have been found to be transported to the cytoplasm to regulate protein at the translational level by associating with miRNAs [88] or ribosomes [89]. To delve deeper into the when and where, RNA imaging tools have been developed for both fixed and live cells [90] (Figure 2). Imaging of fixed cells is based on RNA fluorescence in situ hybridization (FISH), among which single-molecule FISH (smFISH) can realize the imaging of single-molecule RNA and quantify the transcription level and transcript accumulation. Rosa et al. used smFISH to visualize the individual sense and antisense of *FLOWERING LOCUS C* (*FLC*) RNAs in Arabidopsis root, demonstrating that the antisense of *FLC* (*COOLAIR*) transcription is anti-correlated with *FLC* transcription and non-spliced *COOLAIR* accumulates around the *FLC* locus early during the cold [87]. Fixed-cell RNA imaging has enabled in situ study of the transcriptome of tissue samples, which scale up to the genome level [91]. More recently, whole-mount smFISH in several intact plant tissues has been developed to combine RNA and protein quantification at cellular and subcellular resolution by using clearing steps. It is worth mentioning that this simple method was successfully applied to a developing embryo [92]. Moreover, live-cell RNA imaging, including exogenous and endogenous imaging, can provide the critical temporal dimension and precise quantification of RNA molecular dynamics [90]. RNA aptamer Broccoli was employed to examine the subcellular localization of *HID1* in vivo, generating stable transgenic lines expressing *HID* promoter-induced *HID* fused with Broccoli in mutant *hid1* and thus rescuing the delayed germination phenotype of mutant *hid1* [7]. Jiang et al. reported that large Stokes shift fluorescent RNAs can be used to track and quantify multiple RNAs in diverse biological processes [93]. 

Currently, studies have revealed the function of seed lncRNAs by studying the regulatory relationship between miRNA and lncRNA, as well as proteins and lncRNA. For miRNA–lncRNA interactions, lncRNA acts as a pri-miRNA host gene with common promoter regions or a sponge with several miRNA-binding sites functioning in the nucleus. LncRNA also acts as a target mimic in the cytoplasm, which is the general fashion for most interactions. A three-nucleotide bulge in eTM sequences is an anchor to recognize the miRNA binding site in a computational method of sequence pairing, which is expected to eliminate the cleavage function of miRNA on its target [94]. The method has successfully predicted the osa-miR160 and osa-eTM160 interaction, which was verified by using expression experiments and transgenic lines [38]. Mature prediction algorithms and diverse databases have been developed for the mRNA–miRNA interaction. In contrast, the prediction of lncRNA–miRNA is still in the primary stage because it requires the identification, characterization, and naming rule of lncRNA. Experimental verification, in turn, promotes the development of prediction, and direct and high-throughput experiments are required to prove in silico prediction in machine learning approaches. Now, lncRNA-protein interaction has been characterized in vivo and in vitro through RNA-centric methods such as RNA pull-down, RNA binding protein immunoprecipitation (RNA-RIP), and CHIRP-seq (chromatin isolation by RNA purification). To search for potential MISSEN interacting proteins, the result of RNA pull-down with biotinylated MISSEN in developing seed extracts was further confirmed by RIP assay, tRNA-scaffolded Streptavidin Aptamer (tRSA)-RNA pull-down assay, and trimolecular fluorescence complementation (TriFC) [30]. Practically, in vitro methods are definitely effective for determining which nucleotides and amino acids play roles in known RNA–protein interactions, while in vivo methods may be the optimal choice for discovering and analyzing RNA–protein interactions in terms of subcellular localization, RNA and protein modifications, or a dynamic range of local protein concentrations [95]. 

## 5. Future and Challenge

LncRNAs are expressed in a highly tissue-specific or stage-specific manner and are more likely to form high-level structures. Thus, their diverse and highly specific functions is difficult to be determined by using traditional techniques. Although a large amount of non-coding transcript data have been identified and predicted, only a limited number of lncRNAs have confident annotations, let alone mechanistic information in seed biology. LncRNA imaging in situ poses a challenge since embryos are embedded deep in fruit and seed and surrounded by fruit coat and endosperm, especially observing in real-time as lncRNAs are usually expressed at a low level and dynamic changed. RNA contamination from the surrounding tissue and RNA quality are concerns when sampling prior to sequencing. During seed development and germination, the communication among embryo, endosperm, and seed coat is frequent and dynamic. Since the endosperm normal development is essential for seed coat and embryo development, there are too many unanswered questions here as to determining the molecular genetic network of seed formation and germination. For example, how the multi-factors or pathways are coordinated and regulated in endosperm and embryo, how the embryo-derived signals transfer for resource release from the endosperm, what role do lncRNAs play in these processes, and whether the complex interaction between seed longevity and seed dormancy is caused by the multiple lncRNAs in the DOG loci, and what role do lncRNAs play in these processes, and so on. Once endosperm programmed cell death (PCD) is initiated, the endosperm seems to plays a decreasing role regardless of endospermic and non-endospermic seed. The endosperm serves as a storage tissue and protective barrier during seed germination, then how can the lncRNAs be remobilized in these dead cells. When using strategies, it is necessary to pay attention to whether lncRNA is in the promoter region or the 3′-UTR region of the protein-coding gene. In the near future, with the rapid development of artificial intelligence and single-cell genome technology, combining deep learning methods and RNA imaging will make a contribution to our knowledge of seed lncRNAs and have great potential in advancing crop breeding. 

## Data Availability

No new data were created in this study. Data sharing is not applicable to this article.

## References

[1] Commission On Genetic Resources For Food and Agriculture (2011). Strengthening Seed Systems: Gap Analysis of the Seed Sector.

[2] León-Martínez G., Vielle-Calzada J.-P., Grossniklaus U. (2019). Chapter Twenty—Apomixis in flowering plants: Developmental and evolutionary considerations. Current Topics in Developmental Biology.

[3] Lou L., Ding L., Wang T., Xiang Y. (2020). Emerging Roles of RNA-Binding Proteins in Seed Development and Performance. Int. J. Mol. Sci..

[4] Birney E., Stamatoyannopoulos J.A., Dutta A., Guigó R., Gingeras T.R., Margulies E.H., Weng Z., Snyder M., Dermitzakis E.T., Thurman R.E. (2007). Identification and analysis of functional elements in 1% of the human genome by the ENCODE pilot project. Nature.

[5] Chekanova J.A., Gregory B.D., Reverdatto S.V., Chen H., Kumar R., Hooker T., Yazaki J., Li P., Skiba N., Peng Q. (2007). Genome-Wide High-Resolution Mapping of Exosome Substrates Reveals Hidden Features in the Arabidopsis Transcriptome. Cell.

[6] He H., Zhou Y.-F., Yang Y.-W., Zhang Z., Lei M.-Q., Feng Y.-Z., Zhang Y.-C., Chen Y.-Q., Lian J.-P., Yu Y. (2021). Genome-Wide Analysis Identified a Set of Conserved lncRNAs Associated with Domestication-Related Traits in Rice. Int. J. Mol. Sci..

[7] Wang Y., Fan Y., Fan D., Zhou X., Jiao Y., Deng X.W., Zhu D. (2023). The noncoding RNA HIDDEN TREASURE 1 promotes phytochrome B-dependent seed germination by repressing abscisic acid biosynthesis. Plant Cell.

[8] Fang J., Zhang F., Wang H., Wang W., Zhao F., Li Z., Sun C., Chen F., Xu F., Chang S. (2019). Ef-cd locus shortens rice maturity duration without yield penalty. Proc. Natl. Acad. Sci. USA.

[9] Chekanova J.A. (2015). Long non-coding RNAs and their functions in plants. Curr. Opin. Plant Biol..

[10] Daxinger L., Kanno T., Matzke M. (2008). Pol V transcribes to silence. Cell.

[11] Wang W., Min L., Qiu X., Wu X., Liu C., Ma J., Zhang D., Zhu L. (2021). Biological Function of Long Non-coding RNA (LncRNA) Xist. Front. Cell Dev. Biol..

[12] Waseem M., Liu Y., Xia R. (2020). Long Non-Coding RNAs, the Dark Matter: An Emerging Regulatory Component in Plants. Int. J. Mol. Sci..

[13] Ørom U.A., Shiekhattar R. (2013). Long noncoding RNAs usher in a new era in the biology of enhancers. Cell.

[14] Zhu B., Yang Y., Li R., Fu D., Wen L., Luo Y., Zhu H. (2015). RNA sequencing and functional analysis implicate the regulatory role of long non-coding RNAs in tomato fruit ripening. J. Exp. Bot..

[15] Ten Hove C.A., Lu K.-J., Weijers D. (2015). Building a plant: Cell fate specification in the early Arabidopsis embryo. Development.

[16] De Vries S.C., Weijers D. (2017). Plant embryogenesis. Curr. Biol..

[17] Guttman M., Donaghey J., Carey B.W., Garber M., Grenier J.K., Munson G., Young G., Lucas A.B., Ach R., Bruhn L. (2011). lincRNAs act in the circuitry controlling pluripotency and differentiation. Nature.

[18] Bai Y., Liu M., Zhou R., Jiang F., Li P., Li M., Zhang M., Wei H., Wu Z. (2023). Construction of ceRNA Networks at Different Stages of Somatic Embryogenesis in Garlic. J. Integr. Plant Biol..

[19] Chen Y., Li X., Su L., Chen X., Zhang S., Xu X., Zhang Z., Chen Y., XuHan X., Lin Y. (2018). Genome-wide identification and characterization of long non-coding RNAs involved in the early somatic embryogenesis in *Dimocarpus longan* Lour. BMC Genom..

[20] Gao Y., Cui Y., Zhao R., Chen X., Zhang J., Zhao J., Kong L. (2022). Cryo-Treatment Enhances the Embryogenicity of Mature Somatic Embryos via the lncRNA-miRNA-mRNA Network in White Spruce. J. Integr. Plant Biol..

[21] Xiong H., Wang W., Sun M.-X. (2021). Endosperm development is an autonomously programmed process independent of embryogenesis. Plant Cell.

[22] Xu W., Yang T., Wang B., Han B., Zhou H., Wang Y., Li D.Z., Liu A. (2018). Differential expression networks and inheritance patterns of long non-coding RNAs in castor bean seeds. Plant J..

[23] Luo M., Taylor J.M., Spriggs A., Zhang H., Wu X., Russell S., Singh M., Koltunow A. (2011). A Genome-Wide Survey of Imprinted Genes in Rice Seeds Reveals Imprinting Primarily Occurs in the Endosperm. PLoS Genet..

[24] Bauer M.J., Fischer R.L. (2011). Genome demethylation and imprinting in the endosperm. Curr. Opin. Plant Biol..

[25] Raissig M.T., Baroux C., Grossniklaus U. (2011). Regulation and Flexibility of Genomic Imprinting during Seed Development. Plant Cell.

[26] Waters A.J., Makarevitch I., Eichten S.R., Swanson-Wagner R.A., Yeh C.-T., Xu W., Schnable P.S., Vaughn M.W., Gehring M., Springer N.M. (2011). Parent-of-Origin Effects on Gene Expression and DNA Methylation in the Maize Endosperm. Plant Cell.

[27] Zhang M., Xie S., Dong X., Zhao X., Zeng B., Chen J., Li H., Yang W., Zhao H., Wang G. (2014). Genome-wide high resolution parental-specific DNA and histone methylation maps uncover patterns of imprinting regulation in maize. Genome Res..

[28] Jahnke S., Scholten S. (2009). Epigenetic Resetting of a Gene Imprinted in Plant Embryos. Curr. Biol..

[29] Yuan J., Chen S., Jiao W., Wang L., Wang L., Ye W., Lu J., Hong D., You S., Cheng Z. (2017). Both maternally and paternally imprinted genes regulate seed development in rice. New Phytol..

[30] Zhou Y.F., Zhang Y.C., Sun Y.M., Yu Y., Lei M.Q., Yang Y.W., Lian J.P., Feng Y.Z., Zhang Z., Yang L. (2021). The parent-of-origin lncRNA MISSEN regulates rice endosperm development. Nat. Commun..

[31] Bewley J., Bradford K., Hilhorst H., Nonogaki H. (2013). Seeds: Physiology of Development, Germination and Dormancy.

[32] Pinky, Jain R., Yadav A., Sharma R., Dhaka N. (2023). Emerging roles of long non-coding RNAs in regulating agriculturally important seed traits. Plant Physiol. Biochem..

[33] Kim E.D., Xiong Y., Pyo Y., Kim D.H., Kang B.H., Sung S. (2017). Spatio-temporal analysis of coding and long noncoding transcripts during maize endosperm development. Sci. Rep..

[34] Zhu M., Zhang M., Xing L., Li W., Jiang H., Wang L., Xu M. (2017). Transcriptomic Analysis of Long Non-Coding RNAs and Coding Genes Uncovers a Complex Regulatory Network That Is Involved in Maize Seed Development. Genes.

[35] Zhao J., Ajadi A.A., Wang Y., Tong X., Wang H., Tang L., Li Z., Shu Y., Liu X., Li S. (2020). Genome-Wide Identification of lncRNAs During Rice Seed Development. Genes.

[36] Zhao Z., Liu D., Cui Y., Li S., Liang D., Sun D., Wang J., Liu Z. (2020). Genome-wide identification and characterization of long non-coding RNAs related to grain yield in foxtail millet [*Setaria italica* (L.) P. Beauv.]. BMC Genom..

[37] Khemka N., Rajkumar M.S., Garg R., Jain M. (2022). Genome-wide analysis suggests the potential role of lncRNAs during seed development and seed size/weight determination in chickpea. Planta.

[38] Wang M., Wu H.J., Fang J., Chu C., Wang X.J. (2017). A long noncoding RNA involved in rice reproductive development by negatively regulating osa-miR160. Sci. Bull..

[39] Wang Y., Luo X., Sun F., Hu J., Zha X., Su W., Yang J. (2018). Overexpressing lncRNA LAIR increases grain yield and regulates neighbouring gene cluster expression in rice. Nat. Commun..

[40] Huang J., Wang R., Dai X., Feng J., Zhang H., Zhao P.X. (2019). A microRNA biogenesis-like pathway for producing phased small interfering RNA from a long non-coding RNA in rice. J. Exp. Bot..

[41] Ooms J., Leon-Kloosterziel K.M., Bartels D., Koornneef M., Karssen C.M. (1993). Acquisition of Desiccation Tolerance and Longevity in Seeds of *Arabidopsis thaliana* (A Comparative Study Using Abscisic Acid-Insensitive abi3 Mutants). Plant Physiol..

[42] Bentsink L., Jowett J., Hanhart C.J., Koornneef M. (2006). Cloning of DOG1, a quantitative trait locus controlling seed dormancy in Arabidopsis. Proc. Natl. Acad. Sci. USA.

[43] Kowalczyk J., Palusinska M., Wroblewska-Swiniarska A., Pietras Z., Szewc L., Dolata J., Jarmolowski A., Swiezewski S. (2017). Alternative Polyadenylation of the Sense Transcript Controls Antisense Transcription of DELAY of GERMINATION 1 in Arabidopsis. Mol. Plant.

[44] Fedak H., Palusinska M., Krzyczmonik K., Brzezniak L., Yatusevich R., Pietras Z., Kaczanowski S., Swiezewski S. (2016). Control of seed dormancy in Arabidopsis by a cis-acting noncoding antisense transcript. Proc. Natl. Acad. Sci. USA.

[45] Montez M., Majchrowska M., Krzyszton M., Bokota G., Sacharowski S., Wrona M., Yatusevich R., Massana F., Plewczynski D., Swiezewski S. (2023). Promoter-pervasive transcription causes RNA polymerase II pausing to boost DOG1 expression in response to salt. EMBO J..

[46] Cyrek M., Fedak H., Ciesielski A., Guo Y., Sliwa A., Brzezniak L., Krzyczmonik K., Pietras Z., Kaczanowski S., Liu F. (2016). Seed Dormancy in Arabidopsis Is Controlled by Alternative Polyadenylation of DOG1. Plant Physiol..

[47] Thieffry A., Vigh M.L., Bornholdt J., Ivanov M., Brodersen P., Sandelin A. (2020). Characterization of Arabidopsis thaliana Promoter Bidirectionality and Antisense RNAs by Inactivation of Nuclear RNA Decay Pathways. Plant Cell.

[48] Jiang H., Gao W., Jiang B.L., Liu X., Jiang Y.T., Zhang L.T., Zhang Y., Yan S.N., Cao J.J., Lu J. (2023). Identification and validation of coding and non-coding RNAs involved in high-temperature-mediated seed dormancy in common wheat. Front. Plant. Sci..

[49] Sano N., Rajjou L., North H.M., Debeaujon I., Marion-Poll A., Seo M. (2016). Staying Alive: Molecular Aspects of Seed Longevity. Plant Cell Physiol..

[50] Nguyen T.-P., Keizer P., van Eeuwijk F., Smeekens S., Bentsink L. (2012). Natural Variation for Seed Longevity and Seed Dormancy Are Negatively Correlated in Arabidopsis. Plant Physiol..

[51] Waterworth W.M., Footitt S., Bray C.M., Finch-Savage W.E., West C.E. (2016). DNA damage checkpoint kinase ATM regulates germination and maintains genome stability in seeds. Proc. Natl. Acad. Sci. USA.

[52] Waterworth W.M., Latham R., Wang D., Alsharif M., West C.E. (2022). Seed DNA damage responses promote germination and growth in *Arabidopsis thaliana*. Proc. Natl. Acad. Sci. USA.

[53] Châtelain E., Satour P., Laugier E., Ly Vu B., Payet N., Rey P., Montrichard F. (2013). Evidence for participation of the methionine sulfoxide reductase repair system in plant seed longevity. Proc. Natl. Acad. Sci. USA.

[54] Clerkx E.J.M., Blankestijn-De Vries H., Ruys G.J., Groot S.P.C., Koornneef M. (2004). Genetic differences in seed longevity of various *Arabidopsis mutants*. Physiol. Plant..

[55] Mao Z., Sun W. (2015). Arabidopsis seed-specific vacuolar aquaporins are involved in maintaining seed longevity under the control of ABSCISIC ACID INSENSITIVE 3. J. Exp. Bot..

[56] Luo Y., Le J., Zhang Y., Wang R., Li Q., Lu X., Liu J., Deng Z. (2022). Identification and Functional Analysis of LncRNAs in Response to Seed Aging in *Metasequoia glyptostroboides* by Third Generation Sequencing Technology. Forests.

[57] Kiegle E.A., Garden A., Lacchini E., Kater M.M. (2018). A Genomic View of Alternative Splicing of Long Non-coding RNAs during Rice Seed Development Reveals Extensive Splicing and lncRNA Gene Families. Front. Plant Sci..

[58] Zhang Y., Fan F., Zhang Q., Luo Y., Liu Q., Gao J., Liu J., Chen G., Zhang H. (2022). Identification and Functional Analysis of Long Non-Coding RNA (lncRNA) in Response to Seed Aging in Rice. Plants.

[59] Gubler F., Millar A.A., Jacobsen J.V. (2005). Dormancy release, ABA and pre-harvest sprouting. Curr. Opin. Plant Biol..

[60] Penfield S. (2017). Seed dormancy and germination. Curr. Biol. CB.

[61] Wu J., Liu C., Liu Z., Li S., Li D., Liu S., Huang X., Liu S., Yukawa Y. (2019). Pol III-Dependent CabbageBoNR8Long ncRNA Affects Seed Germination and Growth in Arabidopsis. Plant Cell Physiol..

[62] Guo G., Liu X., Sun F., Cao J., Huo N., Wuda B., Xin M., Hu Z., Du J., Xia R. (2018). Wheat miR9678 Affects Seed Germination by Generating Phased siRNAs and Modulating Abscisic Acid/Gibberellin Signaling. Plant Cell.

[63] Ariel F., Jegu T., Latrasse D., Romero-Barrios N., Christ A., Benhamed M., Crespi M. (2014). Noncoding Transcription by Alternative RNA Polymerases Dynamically Regulates an Auxin-Driven Chromatin Loop. Mol. Cell.

[64] Gagliardi D., Cambiagno D.A., Arce A.L., Tomassi A.H., Giacomelli J.I., Ariel F.D., Manavella P.A. (2019). Dynamic regulation of chromatin topology and transcription by inverted repeat-derived small RNAs in sunflower. Proc. Natl. Acad. Sci. USA.

[65] Roulé T., Christ A., Hussain N., Huang Y., Hartmann C., Benhamed M., Gutierrez-Marcos J., Ariel F., Crespi M., Blein T. (2022). The lncRNA MARS modulates the epigenetic reprogramming of the marneral cluster in response to ABA. Mol. Plant.

[66] Tian R., Sun X., Liu C., Chu J., Zhao M., Zhang W.H. (2023). A Medicago truncatula lncRNA MtCIR1 negatively regulates response to salt stress. Planta.

[67] Radoeva T., Vaddepalli P., Zhang Z., Weijers D. (2019). Evolution, Initiation, and Diversity in Early Plant Embryogenesis. Dev. Cell.

[68] Zhang S., Thakare D., Yadegari R. (2018). Laser-Capture Microdissection of Maize Kernel Compartments for RNA-Seq-Based Expression Analysis. Methods Mol. Biol..

[69] Zhan J., Thakare D., Ma C., Lloyd A., Nixon N.M., Arakaki A.M., Burnett W.J., Logan K.O., Wang D., Wang X. (2015). RNA sequencing of laser-capture microdissected compartments of the maize kernel identifies regulatory modules associated with endosperm cell differentiation. Plant Cell.

[70] Kim E., Xiong Y., Kang B.H., Sung S. (2019). Identification of Long Noncoding RNAs in the Developing Endosperm of Maize. Methods Mol. Biol..

[71] Slane D., Kong J., Berendzen K.W., Kilian J., Henschen A., Kolb M., Schmid M., Harter K., Mayer U., De Smet I. (2014). Cell type-specific transcriptome analysis in the early *Arabidopsis thaliana* embryo. Development.

[72] Weinhofer I., Köhler C. (2014). Endosperm-specific chromatin profiling by fluorescence-activated nuclei sorting and ChIP-on-chip. Methods Mol. Biol..

[73] Del Toro-De León G., Köhler C. (2019). Endosperm-specific transcriptome analysis by applying the INTACT system. Plant Reprod..

[74] Palovaara J., Saiga S., Wendrich J.R., van `t Wout Hofland N., van Schayck J.P., Hater F., Mutte S., Sjollema J., Boekschoten M., Hooiveld G.J. (2017). Transcriptome dynamics revealed by a gene expression atlas of the early Arabidopsis embryo. Nat. Plants.

[75] Moreno-Romero J., Santos-González J., Hennig L., Köhler C. (2017). Applying the INTACT method to purify endosperm nuclei and to generate parental-specific epigenome profiles. Nat. Protoc..

[76] Yadav V.K., Santos-González J., Köhler C. (2021). INT-Hi-C reveals distinct chromatin architecture in endosperm and leaf tissues of Arabidopsis. Nucleic Acids Res..

[77] Zheng X.Y., Gehring M. (2019). Low-input chromatin profiling in Arabidopsis endosperm using CUT&RUN. Plant Reprod..

[78] Kao P., Schon M.A., Mosiolek M., Enugutti B., Nodine M.D. (2021). Gene expression variation in Arabidopsis embryos at single-nucleus resolution. Development.

[79] Picard C.L., Povilus R.A., Williams B.P., Gehring M. (2021). Transcriptional and imprinting complexity in Arabidopsis seeds at single-nucleus resolution. Nat. Plants.

[80] Long Y., Liu Z., Jia J., Mo W., Fang L., Lu D., Liu B., Zhang H., Chen W., Zhai J. (2021). FlsnRNA-seq: Protoplasting-free full-length single-nucleus RNA profiling in plants. Genome Biol..

[81] Mo Y., Jiao Y. (2022). Advances and applications of single-cell omics technologies in plant research. Plant J. Cell Mol. Biol..

[82] Li J., Zhang X., Liu C. (2020). The computational approaches of lncRNA identification based on coding potential: Status quo and challenges. Comput. Struct. Biotechnol. J..

[83] Simopoulos C.M.A., Weretilnyk E.A., Golding G.B. (2018). Prediction of plant lncRNA by ensemble machine learning classifiers. BMC Genom..

[84] Cech T.R. (2015). RNA World research-still evolving. RNA.

[85] Crespi M.D., Jurkevitch E., Poiret M., d’Aubenton-Carafa Y., Petrovics G., Kondorosi E., Kondorosi A. (1994). enod40, a gene expressed during nodule organogenesis, codes for a non-translatable RNA involved in plant growth. EMBO J..

[86] Chen L.-L. (2022). Towards higher-resolution and in vivo understanding of lncRNA biogenesis and function. Nat. Methods.

[87] Rosa S., Duncan S., Dean C. (2016). Mutually exclusive sense–antisense transcription at FLC facilitates environmentally induced gene repression. Nat. Commun..

[88] Wang Y., Xu Z., Jiang J., Xu C., Kang J., Xiao L., Wu M., Xiong J., Guo X., Liu H. (2013). Endogenous miRNA Sponge lincRNA-RoR Regulates Oct4, Nanog, and Sox2 in Human Embryonic Stem Cell Self-Renewal. Dev. Cell.

[89] Deforges J., Reis R.S., Jacquet P., Sheppard S., Gadekar V.P., Hart-Smith G., Tanzer A., Hofacker I.L., Iseli C., Xenarios I. (2019). Control of Cognate Sense mRNA Translation by cis-Natural Antisense RNAs. Plant Physiol..

[90] Le P., Ahmed N., Yeo G.W. (2022). Illuminating RNA biology through imaging. Nat. Cell Biol..

[91] Qin T., Xiong L. (2019). Subcellular Localization and Functions of Plant lncRNAs in Drought and Salt Stress Tolerance. Plant Long Non-Coding RNAs.

[92] Zhao L., Fonseca A., Meschichi A., Sicard A., Rosa S. (2023). Whole-mount smFISH allows combining RNA and protein quantification at cellular and subcellular resolution. Nat. Plants.

[93] Jiang L., Xie X., Su N., Zhang D., Chen X., Xu X., Zhang B., Huang K., Yu J., Fang M. (2023). Large Stokes shift fluorescent RNAs for dual-emission fluorescence and bioluminescence imaging in live cells. Nat. Methods.

[94] Wu H.-J., Wang Z.-M., Wang M., Wang X.-J. (2013). Widespread Long Noncoding RNAs as Endogenous Target Mimics for MicroRNAs in Plants. Plant Physiol..

[95] Ramanathan M., Porter D.F., Khavari P.A. (2019). Methods to study RNA-protein interactions. Nat. Methods.

